# Automation of electrothermal cell sheet manipulator for seamless tissue assembly and handling

**DOI:** 10.1007/s10544-025-00781-y

**Published:** 2025-11-21

**Authors:** Sehong Kang, Min Ku Kim, Chi Hwan Lee, Hyunjoon Kong

**Affiliations:** 1 Department of Mechanical Science and Engineering, University of Illinois at Urbana-Champaign, Urbana, USA; 2 Department of Chemical and Biomolecular Engineering, University of Illinois at Urbana-Champaign, Urbana, USA; 3 Chan Zuckerberg Biohub Chicago, Chicago, USA; 4 Scott H. Fisher Multicellular Engineered Living Systems Theme, Carl R. Woese Institute for Genomic Biology, University of Illinois at Urbana-Champaign, Urbana, USA; 5 Biomedical and Translational Sciences, Carle Illinois College of Medicine, University of Illinois at Urbana-Champaign, Urbana, USA; 6 Weldon School of Biomedical Engineering, Purdue University, West Lafayette, USA; 7 School of Mechanical Engineering, Purdue University, West Lafayette, USA; 8 School of Materials Engineering, Purdue University, West Lafayette, USA; 9 Birck Nanotechnology Center, Purdue University, West Lafayette, USA; 10 Elmore Family School of Electrical and Computer Engineering, Purdue University, West Lafayette, USA; 11 School of Mechanical Engineering, Hanyang University, Seoul, Seoul, Republic of Korea

**Keywords:** Human induced pluripotent stem cell, Human brain microvascular endothelial cell, Thermoresponsive hydrogel, Biofabrication, Automated biofabrication, Compliance-based z-axis apparatus

## Abstract

The manipulation of fragile biological tissues such as engineered cell sheets remains a major challenge for regenerative medicine and tissue engineering. Manual handling with tools like tweezers often induces wrinkling or tearing, compromising tissue integrity. Here, we present an automated cell sheet manipulator that integrates a thermoresponsive microchanneled poly(N-isopropylacrylamide) (PNIPAAm) hydrogel with an embedded microheater, mounted on a programmable three-axis motorized stage. Upon localized heating and cooling, the hydrogel undergoes rapid, reversible volumetric transitions that enable suction-based gripping and release of cell sheets within a few seconds. The custom LabVIEW interface synchronizes stage movement and thermal cycling, allowing reproducible, hands-free operation. A compliance-based Z-axis apparatus ensured uniform low-magnitude contact forces, preventing mechanical damage during transfer. Using this system, human iPSC-derived neural sheets were reliably transferred onto human brain microvascular endothelial cell (hBMEC) monolayers. Compared to manual transfer, the automated manipulator preserved cell sheet flatness and minimized micro-wrinkling, resulting in safe retention of intercellular architecture and structural integrity. This work demonstrates a robust, user-friendly platform for automated and gentle handling of delicate biological sheets. By enabling the precise stacking of engineered tissues while preserving their morphology, this system provides a promising tool for advanced biofabrication workflows, supporting defect-free 3D tissue assembly and implantation.

**Supplementary Information:**

The online version contains supplementary material available at 10.1007/s10544-025-00781-y.

## Introduction

A cell sheet is a thin, continuous layer of cells forming a cohesive tissue-like construct. Cell sheet technology enables the fabrication of contiguous layers of cells with an intact extracellular matrix, preserving cell surface proteins and cell–cell junctions (Zurina et al. 2023). This approach allows for creation of functional, transplantable tissues that closely mimic native tissue architecture and function. Cell sheet engineering has been successfully applied in regenerative medicine for a variety of tissues and organs, including heart (Furuta et al. 2006; Ott et al. 2008), skin (Takahashi et al. 2011), and liver (Ohashi et al. 2007). For example, stem cell-derived cell sheets have emerged as promising tools for regenerative therapies, where success often hinges on the ability to place these delicate sheets onto target tissues without deformation or rupture, and to ensure robust engraftment to host tissues (Lin et al. 2013; Cerqueira et al. 2013; Ryu et al. 2020). Beyond transplantation, cell sheet technology is also being explored for constructing three-dimensional tissue models and for applications in drug discovery and disease modeling (Sasagawa et al. 2010; Takahashi et al. 2011). However, the fragile, hydrated, and ultra-thin nature of these biological substrates makes them prone to tearing, folding, or detachment during manual manipulation (Zurina et al. 2023).

The precise handling and transfer of engineered cell sheets and primary tissue slices remain critical yet unresolved challenge across numerous biomedical applications in both fundamental and translational studies (Kobayashi et al. 2019; Zurina et al. 2023). Conventional handling techniques, including temperature-responsive culture substrates, sacrificial layers, and polymeric support films, require additional steps for sheet retrieval and release. These steps increase procedural complexity, and introduce risks of contamination and mechanical stress (De et al. 2007; Zurina et al. 2023). To overcome these limitations, soft robotics and hydrogel-based interfaces have gained attention due to their mechanical compliance (Lee et al. 2016; Kobayashi et al. 2019; Ambhorkar et al. 2020). However, many of these systems were transferred gel-scaffolded tissue models and suffer from long actuation times, poor durability in wet environments, and limited dynamic control of adhesion. These shortcomings are particularly problematic in settings demanding repeated, precise, and automated operation.

Moreover, the integration of such technologies into robotic automation platforms remains largely underexplored, impeding their use in standardized workflows for clinical or laboratory settings (Tadakuma et al. 2013; Maeda et al. 2015; Leangarun et al. 2016; Osada et al. 2020; Ambhorkar et al. 2020). We previously introduced a soft, thermo-responsive, hydrogel-based manipulator featuring embedded microchannels that enable reversible adhesion via electrothermally modulated suction (Kim et al. 2020). This feature could be achieved without the need for external pumps or adhesives. This manipulator enabled manual attachment and release of delicate samples, such as silicon wafers, glass coverslips, and cell sheets, within 20 s. While effective, its manual operation constrained throughput and reproducibility, limiting its broader applicability.

In this study, we advanced this electrothermal manipulator by integrating it into a fully automated platform designed for reliable, repeatable, and user-friendly manipulation of delicate biological tissues. The system combines the hydrogel-based manipulator with a three-axis motorized stage, a LabVIEW-based graphical user interface (GUI), and a real-time control sequence. To demonstrate its performance, we used human induced pluripotent stem cell (hiPSC)-derived neural sheets and human brain microvascular endothelial cell (hBMEC) monolayers as representative fragile tissue models. The integrated platform offers a promising solution for applications in regenerative medicine, such as 3D tissue assembly and automated cell sheet implantation, as well as fundamental research requiring consistent handling of primary tissue slices.

## Methods

### Fabrication of thermoresponsive hydrogel and manipulator

The fabrication of the microchanneled hydrogel followed a previously reported method with slight modifications (Lee et al. 2015). Briefly, N-isopropylacrylamide (NIPAAm, 1.25 g) and the crosslinker N, N’-methylenebisacrylamide (0.01 wt%) were dissolved in distilled water (8.75 ml) at 25 °C for 24 h. A photoinitiator (Irgacure 2959, 0.5 wt%) was then added and fully dissolved. The pre-gel solution was poured into a silicone mold placed on a Si-wafer substrate. For directional crystallization, the assembly was positioned 1 cm above a liquid nitrogen surface. After complete crystallization, the sample was cryo-polymerized by UV irradiation (λ = 365 nm) for 6 h at − 20 °C. The resulting PNIPAAm hydrogel was thoroughly washed with water to remove ice templates. Hydrogel disks were then punched to size and adhered to a microheater using cyanoacrylate adhesive at room temperature.

### Characterization of hydrogel

The morphology of the microchanneled hydrogel was examined after freeze-drying. Samples were immersed in liquid nitrogen for 10 s and subsequently lyophilized to preserve the microchannel structure. The dried hydrogels were then imaged using a field-emission environmental scanning electron microscope (ESEM-FEG; FEI). Thermal response was evaluated by monitoring temperature changes of both the embedded microheater and the microchanneled hydrogel using an infrared camera (E40; FLIR Systems).

### Fabrication of flexible microheater

The microheater was fabricated starting from a copper–polyimide laminate substrate (9 μm Cu/12 μm PI; Pyralux AC091200EV, DuPont). The copper layer was patterned into a Joule heating element through standard photolithography using a dry-film resist (Riston MM540, DuPont), followed by wet chemical etching (CE-100, Transene) to define the conductive traces. To prevent oxidation during high-temperature operation under humid conditions, the exposed copper surface was coated with a 1 μm tin layer using an immersion tin solution (421 Liquid Tin, MG Chemicals). The completed microheater was then powered by an external voltage source (DC Power supply, Dr. Meter).

### Assembly of motorized automatic cell sheet manipulator and design of user interface

The Z-axis location control apparatus and manipulator plunger were designed using CAD software and fabricated with a 3D printer (UltiMaker 2; UltiMaker). The motorized platform was modified from a commercial three-axis engraving stage, and the custom Z-axis apparatus with the manipulator was mounted on the stage’s motor plate. Three stepper motors were controlled by a GRBL board connected to a computer through an NI DAQ interface. The microheater embedded in the manipulator was connected to power generators via a relay and linked to the computer through Arduino and NI DAQ. Both heating and stage motion were controlled through a custom LabVIEW graphical user interface (GUI) using serial communication.

### Force measurement

The force applied to the culture dish was measured with and without the Z-location apparatus using two types of 60-mm tissue culture dishes (Falcon standard tissue culture dish and Thermo Scientific BioLite cell culture dish). A force sensor (SparkFun Electronics) was positioned on the culture dishes to measure the force applied as the manipulator was lowered onto the surface. The system was calibrated using standard weights prior to measurement. The collected data were analyzed to compare the force profiles of the manipulator operated with and without the Z-location apparatus across the two types of culture dishes.

### Cell culture maintenance

Human iPSC-derived neural stem cells (hiNSCs; Cell Applications Inc.) were cultured on glass coverslips coated with Matrigel (Corning). Cells were maintained in neural expansion medium consisting of Dulbecco’s Modified Eagle Medium(DMEM; Thermo Fisher Scientific) supplemented with 20 ng/mL epidermal growth factor (EGF; R&D systems), 20 ng/ml basic fibroblast growth factor (bFGF; R&D systems), and B-27 Supplement without vitamin A (Thermo Fisher Scientific). Neural differentiation was induced for 14 days in Neurobasal medium (Thermo Fisher Scientific) supplemented with B-27 Supplement, N-2 Supplement (Thermo Fisher Scientific), GlutaMAX (Thermo Fisher Scientific), and 10 ng/ml of brain-derived neurotrophic factor (BDNF; ACROBiosystems). After differentiation, cells were maintained in neural maintenance medium consisting of Neurobasal Medium supplemented with B-27 and GlutaMax until use. GFP-expressing human brain microvascular endothelial cells (hBMECs; Angio-Proteomie) were cultured in Endothelial Growth Medium (EGM; Angio-Proteomie) according to the manufacturer’s instructions. Confluent hBMEC monolayers were used for subsequent co-culture assembly. All cells were cultured at 37 °C in a humidified incubator with 5% CO_2_.

### Fabrication of cell sheets and cell sheet multilayers

hiNSC sheets were detached from coverslips by gently scraping the edges with a pipette tip and tapping the culture plate until spontaneous release occurred. The detached hiNSC sheets were then transferred onto confluent hBMEC monolayers using the automated manipulator. The co-cultures were maintained in a 1:1 mixture of EGM and neural maintenance medium for three days before immunostaining. For micro-wrinkling analysis, differentiated hiNSC sheets were fixed and transferred onto glass slides either manually using tweezers or with the automated manipulator.

### Fluorescent imaging of cell sheets

Cell sheets were fixed with 4% paraformaldehyde (Sigma) in 1× Dulbecco’s Phosphate-Buffered Saline (DPBS; Thermo Fisher) for 30 min at room temperature. Fixed samples were permeabilized with 0.1% Triton X-100 (Sigma) for 10 min, followed by blocking with 2% bovine serum albumin (Sigma) for 1 h. For hBMEC/hiNSC multilayers, primary antibody staining was performed with chicken anti-GFAP (1:4000, HelloBio) for 3 h at room temperature. For hiNSC sheets, mouse anti-βIII tubulin (Tuj1; 1:4000, HelloBio) and chicken anti-GFAP (1:4000, HelloBio) were used under the same conditions. After DPBS washes, samples were incubated with the appropriate secondary antibodies for 2 h at room temperature: goat anti-mouse Alexa Fluor 488 (Abcam) and donkey anti-chicken Alexa Fluor 647 (Abcam). Nuclei were counterstained with DAPI (Fluoromount-G mounting medium with DAPI, Invitrogen). For imaging, GFP-hBMEC/hiNSC multilayers were captured using a stereo zoom microscope (Axio Zoom.V16, ZEISS) to visualize large fields of view. hiNSC sheets transferred either by tweezers or by the automated manipulator were imaged using a confocal microscope (LSM-900, ZEISS) to evaluate micro-wrinkling morphology.

## Results

### System overview of the automated cell sheet manipulator

**Fig. 1:**
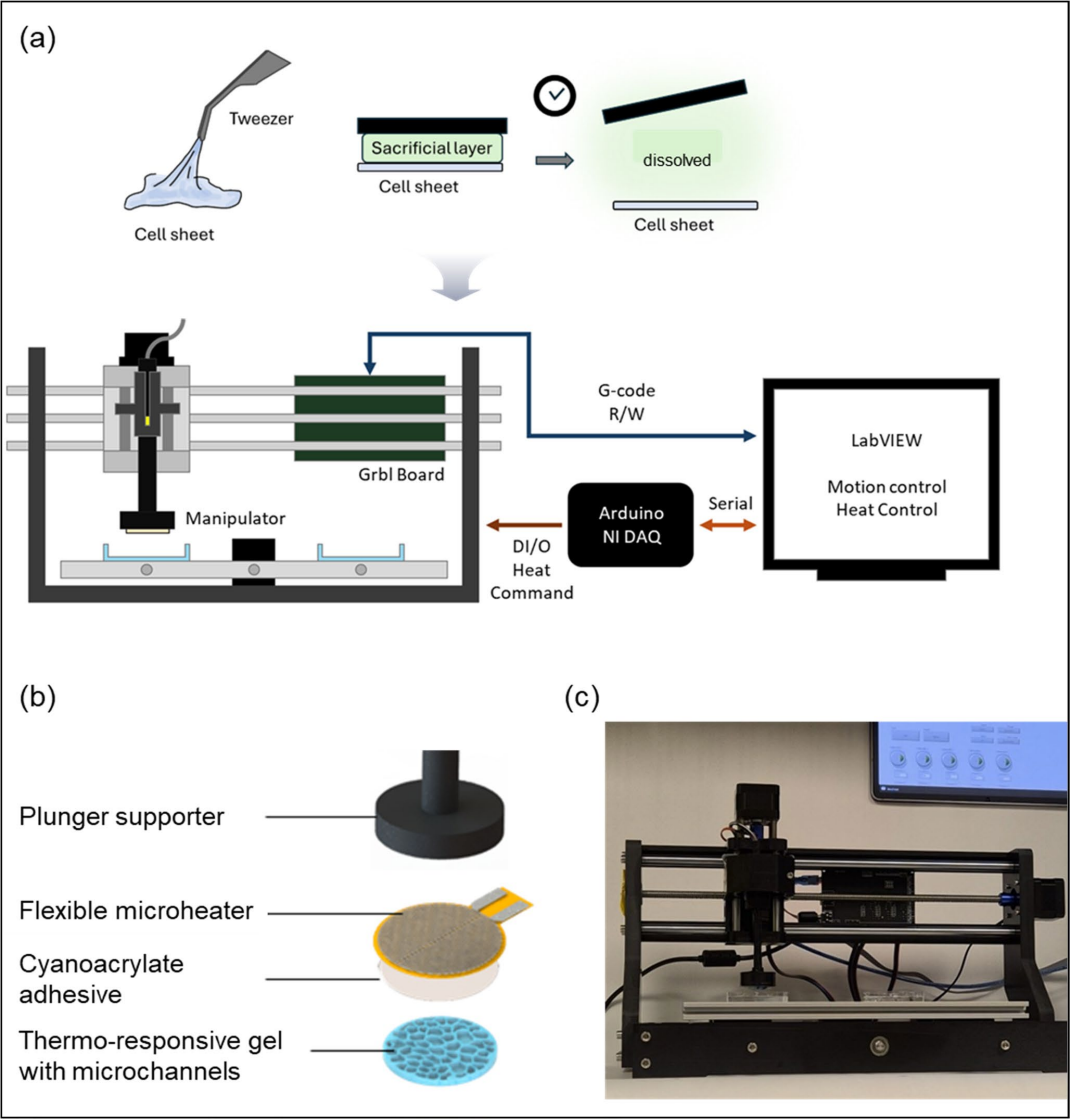
Overview of the automated cell sheet manipulator system. (**a**) Schematic illustration of conventional cell sheet transferring method and the setup showing the manipulator mounted on a motorized 3D stage, controlled via a Grbl board and Arduino/NI DAQ interface, with LabVIEW for motion and temperature control. (**b**) Exploded view of the manipulator components, consisting of a plunger supporter, flexible microheater, adhesive layer, and thermo-responsive gel with microchannels. (**c**) Photograph of the assembled device alongside the LabVIEW control interface

The automated electrothermal manipulator was designed to enable transfer of frail, thin cell sheets through the integration of mechanical, thermal, and control systems (Fig. 1). The manipulator is mounted on a motorized three-axis stage that provides precise positioning in the X, Y, and Z directions. Motion control is coordinated by a Grbl board connected to an Arduino/NI DAQ interface, with commands generated and executed via LabVIEW. This configuration allows programmable manipulation routines through G-code instructions, enabling reproducible movements and automated gripping/releasing cycles.

The manipulator consists of a thermoresponsive hydrogel disk containing aligned microchannels adhered to an embedded microheater (Fig. 1b). The microheater is directly connected to the control system via serial communication, allowing for real-time heat control. The microheater regulates local temperature at the gel surface, which in turn controls gripping and releasing of the cell sheet through reversible water content change in the hydrogel. All components are assembled into a compact device that is interfaced with the LabVIEW control panel, providing user-defined control of stage motion and heating (Fig. 1c). The control system synchronizes microheater activation with manipulator movement to enable precise gripping and release, ensuring accurate placement of the cell sheet at the target location. This integration of hardware and software components facilitates robust, automated cell sheet handling with minimal operator intervention for advanced biofabrication (Fig. 1c).

### Fabrication and characterization of the thermosresponsive microchanneled hydrogel

**Fig. 2:**
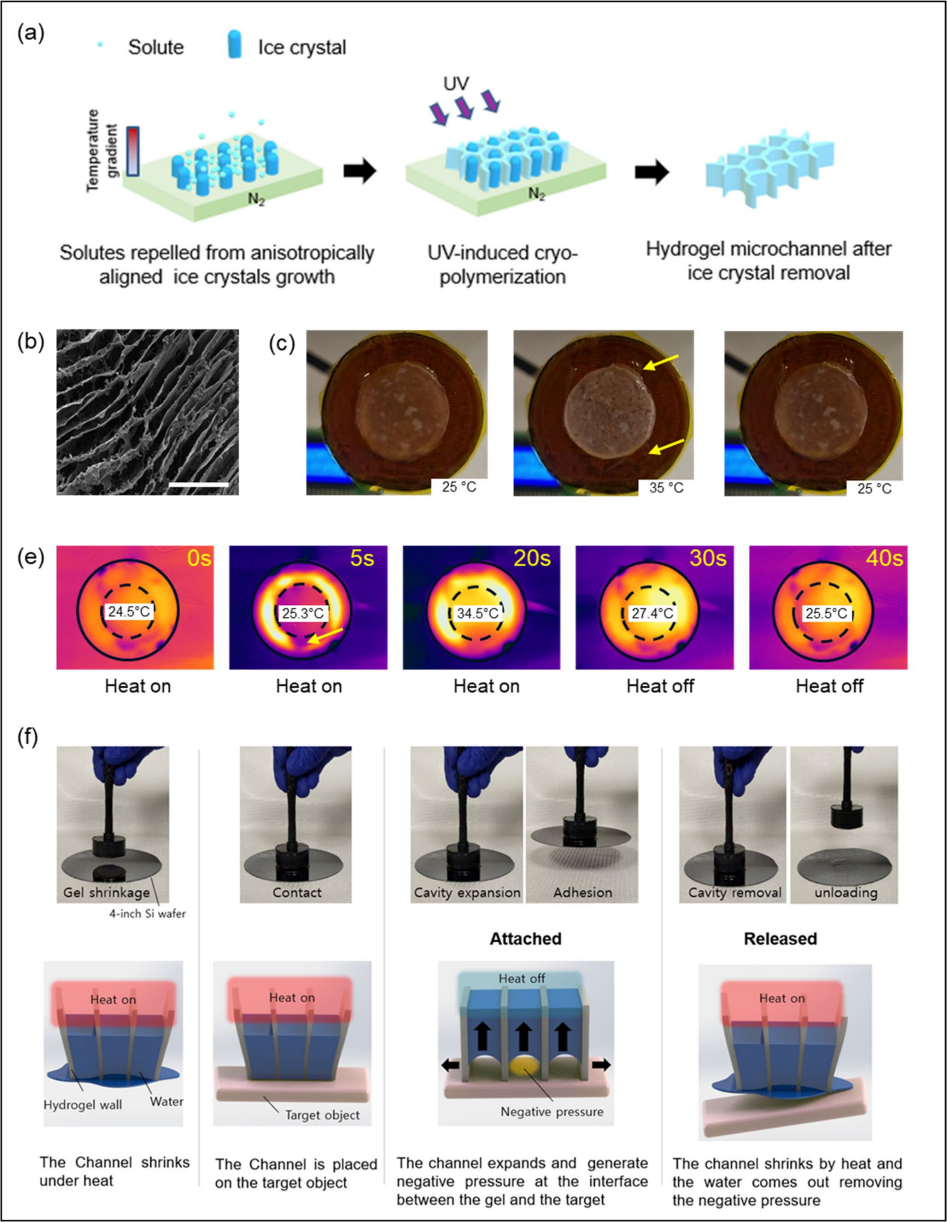
Fabrication and characterization of the thermoresponsive microchanneled hydrogel. (**a**) Schematic of the freeze-templated fabrication process: directional ice crystal growth during freezing, UV-initiated cryo-polymerization, and formation of aligned microchannels after ice crystal removal. (**b**) SEM image showing aligned microchannels (scale bar = 50 μm). (**c**) Photographs of the hydrogel at 25 °C (heat off), 35 °C (heat on), and 25 °C (heat off), demonstrating thermally induced shrinkage and recovery. (**d**) Thermal camera images showing heating dynamics after turning the microheater attached to the gel on and off. Solid black circle outlines the manipulator, dashed black circle outlines the microchanneled gel, and yellow arrows indicate water smeared out of the gel. The indicated temperatures correspond to the center of the microchanneled gel (**e**) Snapshots showing that the manipulator can grip and release a silicon wafer in response to on and off of the microheater. (f) Schematic illustration depicting the change in pressure in microchannels in response to temperature change

To enable controlled gripping and releasing of cell sheets, we fabricated a ultra-thermoresponsive poly(N-isopropylacrylamide) (PNIPAAm) hydrogel with aligned microchannels (Fig. 2). As previously report (Lee et al. 2015), the pre-gelled aqueous solution containing N-isopropylacrylamide (NIPAAm, monomer), N,N’-methylenebisacrylamide (cross-linker), and Irgacure 2959 (photo-initiator) was loaded into a silicone mold and subjected to directional crystallization by positioning it above a liquid nitrogen surface (Fig. 2a). During freezing, anisotropic ice crystal growth induced ordered voids, while solutes were excluded into the interstitial spaces. Subsequent cryo-polymerization under UV irradiation at −20 °C fixed the network, and removal of ice templates through water washing yielded a PNIPAAm gel with aligned microchannels.

The fabricated hydrogel exhibited a uniform geometry, and scanning electron microscopy confirmed the presence of aligned channels (Fig. 2b). This microchanneled architecture promotes rapid water transport and enables efficient volumetric changes during thermal cycling. Functional evaluation showed fast and reversible actuation in response to temperature changes. The gel shrank upon heating from 25 °C to 35 °C and re-expanded upon cooling from 35 °C to 25 °C, completing the transition within 20 s (Fig. 2c). Thermal imaging further revealed the dynamic heating profile of the hydrogel (Fig. 2d). The integrated microheater reached 35 °C within 5 s after activation, while the hydrogel surface achieved the same temperature in ~ 20 s. Upon deactivation, both the microheater and hydrogel returned to 25 °C within 20 s under ambient air conditions.

**Fig. 3:**
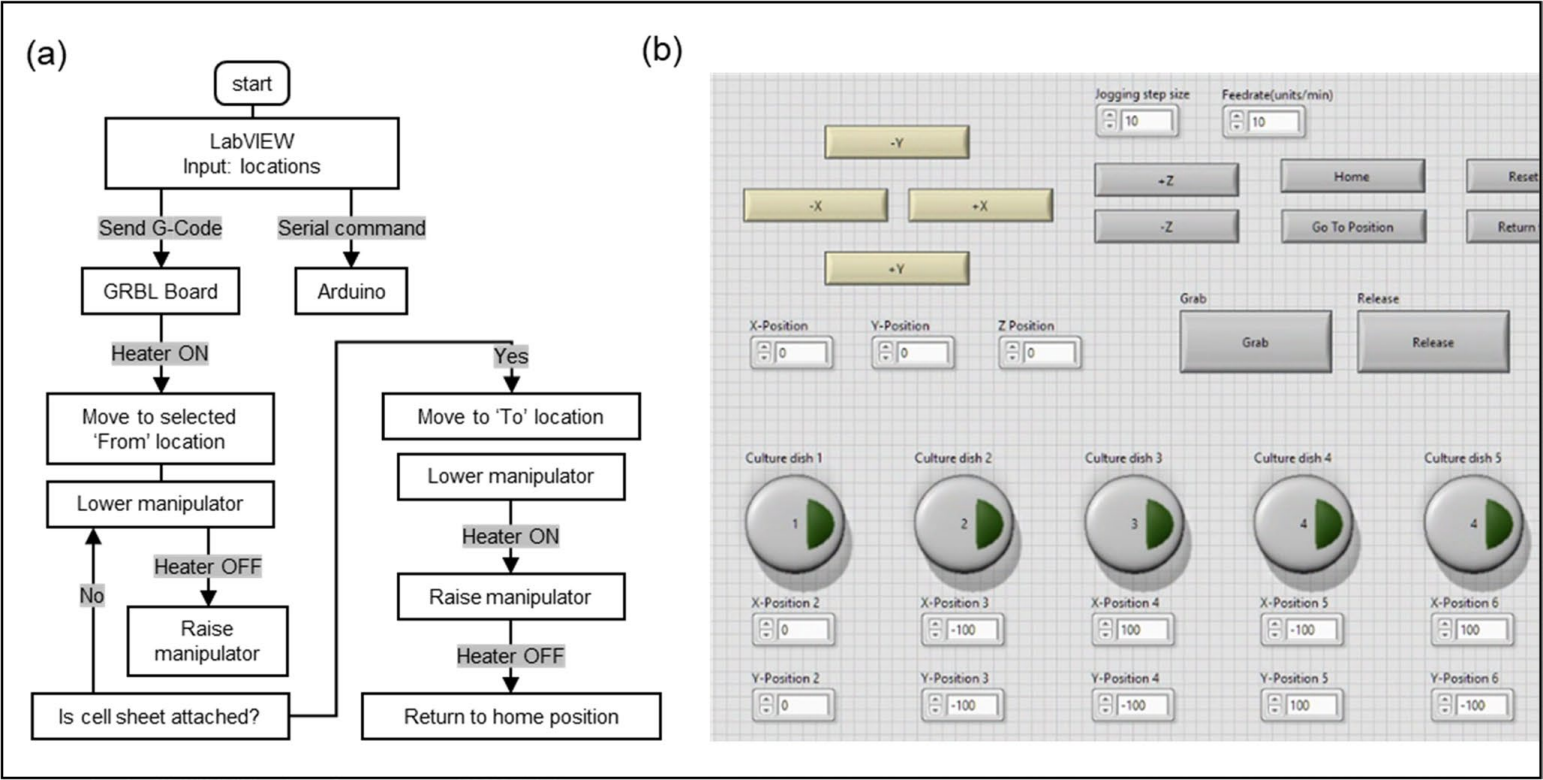
Control workflow and LabVIEW interface for automated cell sheet manipulation. (**a**) Flow chart of the gripping and releasing algorithm, including stage-motion sequences and thermal activation steps. (**b**) LabVIEW graphical user interface for controlling stage movement, manipulator actuation, and heating parameters

Such rapid and reversible actuation enabled controlled gripping and release of various sheets using the manipulator system. When the microheater is activated, the hydrogel channels shrink and expel water as the manipulator approaches the target substrate (Fig. 3e, f). Upon contact with the substrate and subsequent deactivation of the microheater, the upper region of the channels begins to expand by attracting water, generating a negative pressure between the hydrogel and the target surface. This suction force enables the manipulator to grip the substrate. To release it, heat is reapplied to induce hydrogel shrinkage and water expulsion, thereby eliminating the negative pressure and allowing the substrate to detach.

### Control strategy and automation workflow

The movement of the cell sheet manipulator is controlled through a LabVIEW-based program, as illustrated in Fig. 3a. The user interface (Fig. 3b) allows selection of target gripping and releasing locations, which are converted into G-code instructions and transmitted to the microprocessor of a 3-axis motion stage to direct manipulator positioning. Each culture dish was mechanically fixed relative to the bottom axis of the motorized stage, ensuring consistent positional alignment throughout the experiment. The FROM and TO locations correspond directly to these fixed dish positions, and thus no additional image-based or sensor-based calibration was used. At the designated gripping location, the control sequence is configured to activate the Z-axis stepper motor and engage the microheater relay through its control unit. Feedback mechanism from the user interface determines whether to repeat the gripping cycle or advance to the release phase. Upon confirmation, the manipulator is programmed to move to the target release location at a predefined jogging speed. The release process mirrors the gripping process, involving synchronized activation of the Z-axis motor and microheater relay to enable cell sheet detachment. After release, the manipulator is set to return automatically to the home hydration position and await the next execution cycle.

### Mechanical design of the z-location apparatus for precise positioning

**Fig. 4:**
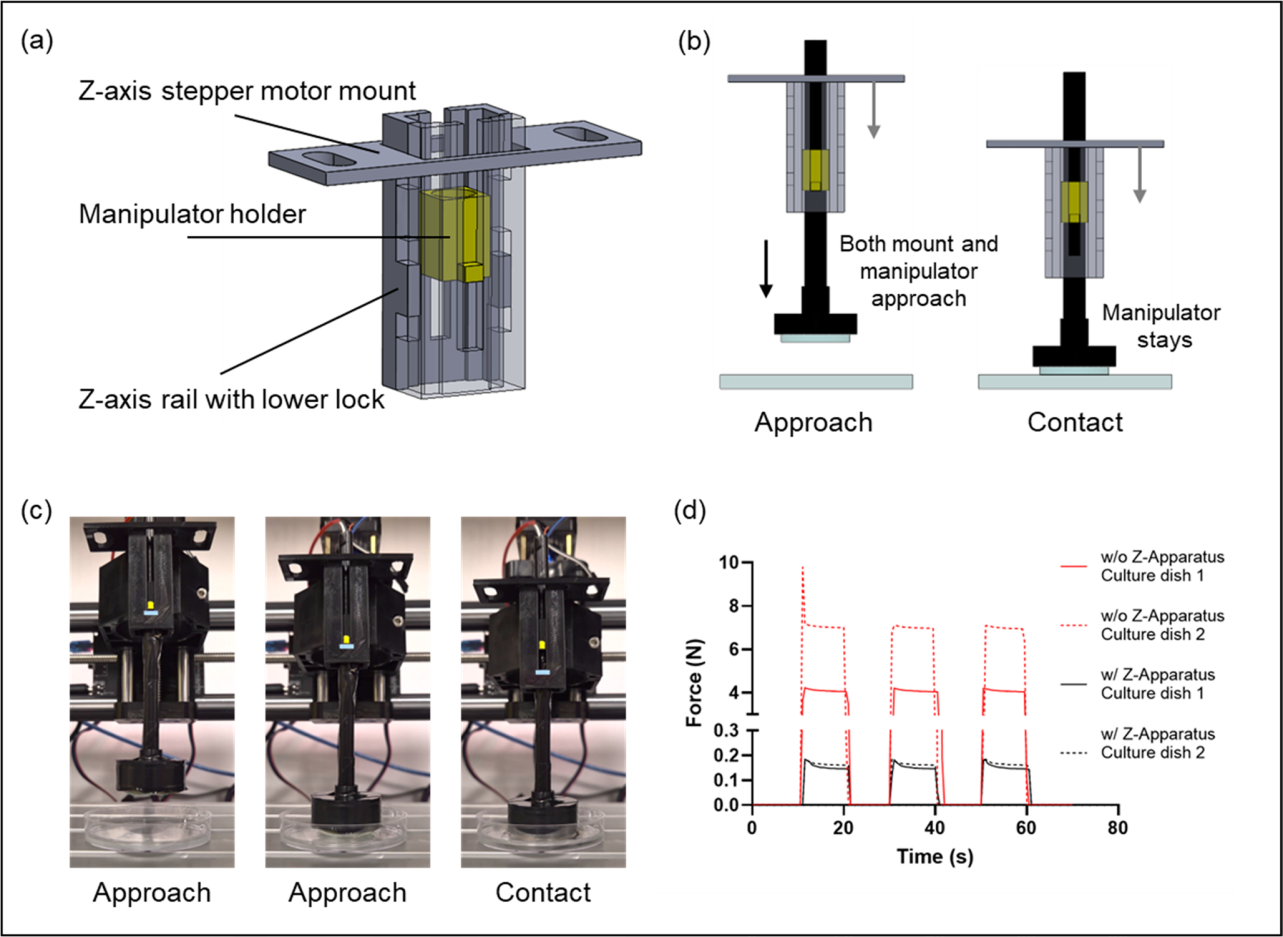
Design and operation of the z-location apparatus for precise vertical control of the manipulator. (**a**) CAD model highlighting the stepper-motor mount, manipulator holder, Z-axis rail, and lower lock. (**b**) CAD renderings illustrating programmed Z-positioning during approach and contact. (**c**) Sequential images showing controlled vertical motion of the manipulator during operation. (**d**) Change of applied force during cell sheet manipulation on different types of culture dish. Red: without z-apparatus, black: with z-apparatus for solid line: culture dish 1 and dashed line: culture dish 2

Precise control of the Z-axis position is essential for manipulating delicate cell sheets without causing damage. Consistent and low-magnitude contact force is essential for preserving the integrity of fragile cell sheets, as it minimizes local mechanical stress and prevents tearing or deformation during manipulation. However, variability in sheet thickness and culture wares produce a challenge for maintaining uniform force during contact. To overcome this, we developed a Z-location apparatus that decouples vertical positioning from direct motor drive, introducing passive compliance and ensuring that a uniform load is applied to the cell sheet surface (Fig. 4a).

The apparatus consists of a fixed stepper motor mount, a Z-axis rail with a lower locking mechanism, and a manipulator holder integrated into the manipulator body (Fig. 4b). During XY translation, the manipulator is secured by the lower lock, maintaining stability. Once positioned above the target location, the manipulator translates freely along the vertical rail as it approaches the cell sheet. Because the Z-axis is unconstrained at this stage, the manipulator makes contact under its controlled load, eliminating direct torque transmission from the stepper motor and ensuring a uniform, low-magnitude load.

Sequential optical images confirmed stable and controlled Z-axis displacement during manipulation (Fig. 4c), and the corresponding vertical motion and contact behavior can be observed in real time in the supplementary video (Video S1). Force monitoring also demonstrated that the apparatus effectively regulated contact force, preventing excessive pressure on the cell sheet during gripping and release cycles with different types of culture ware (Fig. 4d). This result indicates that the Z-apparatus not only reduces the overall magnitude of the applied force but also maintains a uniform load across different substrate types. This compliance-based design ensures consistent pressure application and reduces the risk of damaging cell sheets, even in variety in cells sheets or culture wares.

###  Demonstration of the automated cell sheet manipulator

**Fig. 5:**
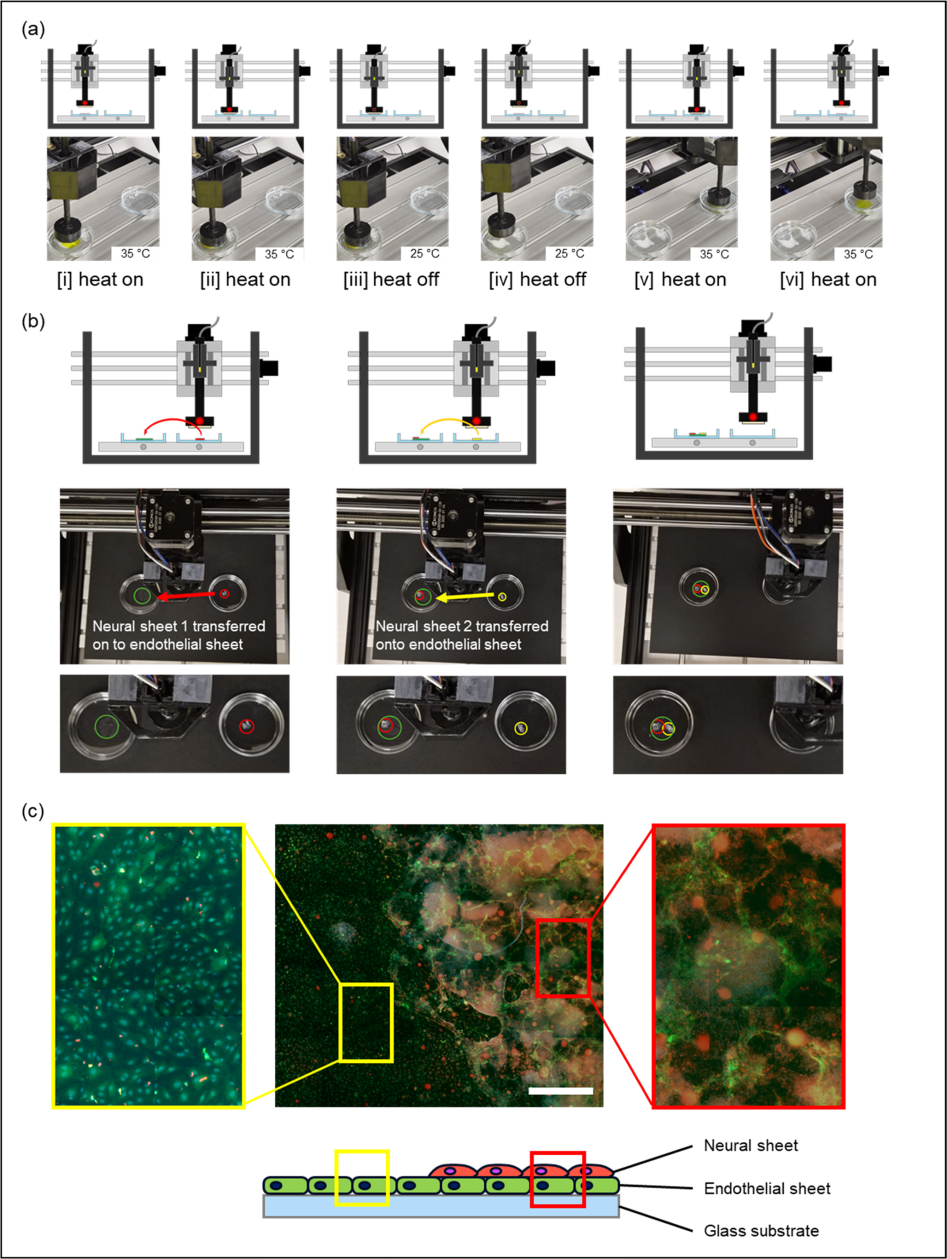
Automated process of cell sheet transfer using the automated cell sheet manipulator. (**a**) Stepwise illustration of the gripping and releasing cycle: (i) heat applied to release water from the hydrogel while the manipulator moves downward, (ii) contact with the cell sheet, (iii) heat switched off to generate negative pressure and grip the cell sheet, (iv) transfer of the gripped sheet to the target location, (v) heat reapplied to release the sheet, and (vi) manipulator lifted away while the hydrogel is rehydrated to prepare for the next cycle. (**b**) Demonstration of sequential transfer of hiPSC-derived neural cell sheets (red circle: neural sheet 1, yellow circle: neural sheet 2) onto a human brain microvascular endothelial cell (hBMEC) monolayer (green circle). Arrows indicate the direction of sheet movement during transfer. (**c**) Confocal image of an hBMEC monolayer after overlay with the hiPSC-derived neural sheet using the manipulator. Scale bar: 200µm. The astrocyte-specific glial fibrillary acidic protein (GFAP, red), and GFP from hBMEC are shown in images

The automated electrothermal manipulator operates through a programmed sequence of heating and cooling steps synchronized with stage positioning to achieve gripping, transfer, and release of cell sheets (Fig. 5). The stepwise gripping and releasing sequence are illustrated in Fig. 5a and Video S2. Upon activation, heating to 35 °C induced hydrogel shrinkage and expulsion of water from the channels, while the manipulator moved downward to approach the target sheet (Step i). While heated, the manipulator made brief contact with the cell sheet and held for one second to establish surface conformity (Step ii). Cooling the hydrogel back to 25 °C created a negative pressure within the channels, drawing water into the matrix and initiating capillary-driven gripping of the sheet (Step iii). With the sheet secured, the manipulator was repositioned to the transfer location (Step iv). Heating was then reapplied, reversing the pressure differential and detaching the cell sheet (Step v). The manipulator lifted away while still heated (Step vi) and finally returned to the home position with heating off, in preparation for the next cycle (Step vii).

To demonstrate functionality in a biological setting, hiPSC-derived neural sheets were transferred onto a human brain microvascular endothelial cell (hBMEC) monolayer (Fig. 5b). The system was also capable of sequentially depositing multiple neural sheets, as highlighted by the transfer of a second sheet onto the same endothelial layer. When transferred using the automated cell sheet manipulator, hiPSC-derived neural sheets maintained close contact with the endothelial layer, forming a continuous and uniform overlay (Fig. 5c). Higher magnification images confirmed that the sheets remained flat, with minimal folding or disruption. Notably, regional differences were observed in endothelial morphology depending on neural sheet coverage, suggesting that transferred sheets can directly interact with the underlying cell layer.

**Fig. 6:**
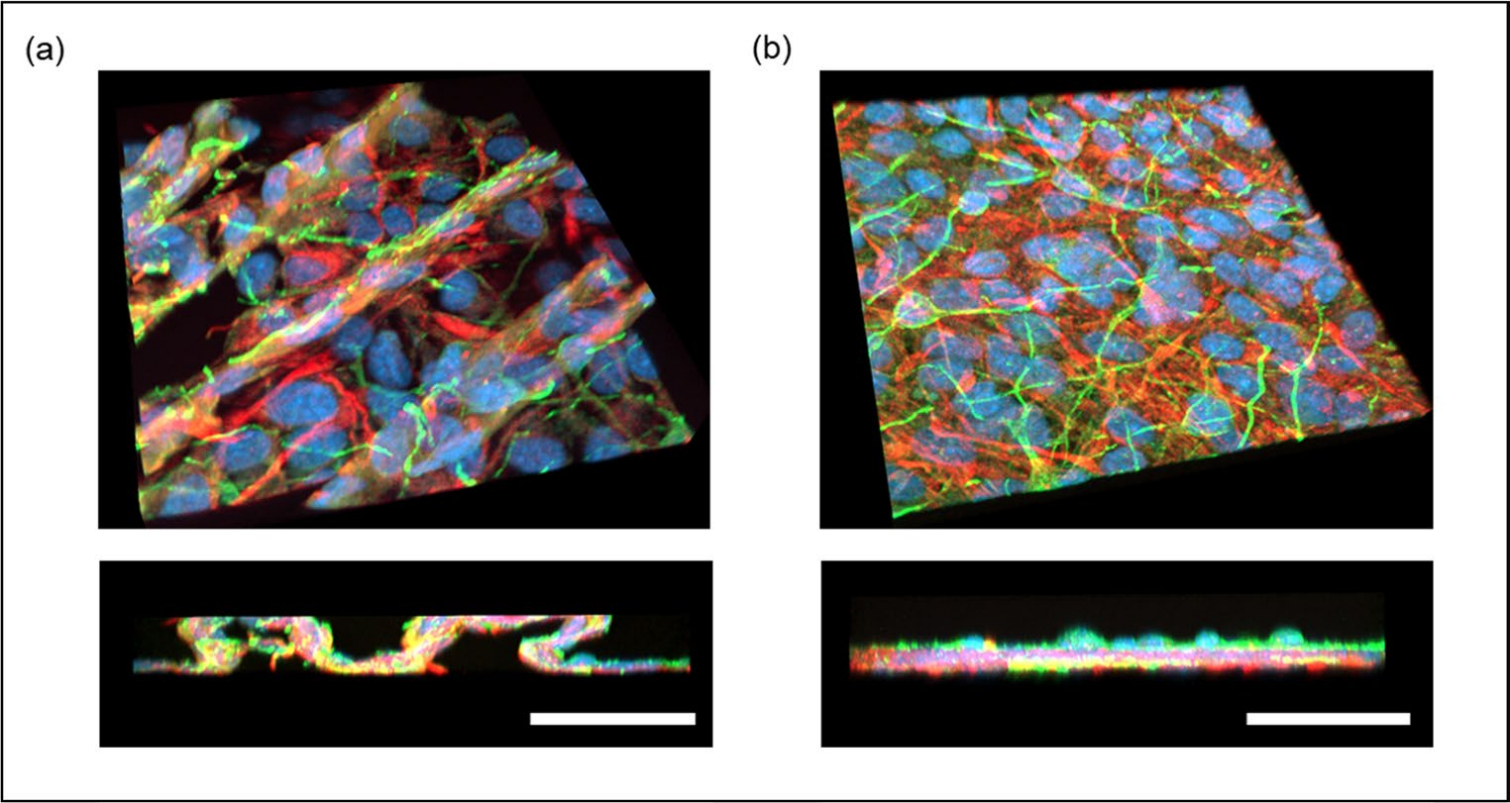
Immunofluorescent imaging of transferred neural cell sheets. (**a**) Sheet transferred manually using tweezers exhibited localized folding and micro-wrinkling. (**b**) Sheet transferred using the automated manipulator maintained a flat morphology without micro-wrinkling. scale bar: 50 μm. The neuronal biomarker β-III tubulin (Tuj1, green), the astrocyte-specific glial fibrillary acidic protein (GFAP, red), and DAPI (blue) are shown in images

The performance of the manipulator was further compared with conventional manual transfer using tweezers (Fig. 6a). Neural sheets transferred manually exhibited localized folding and micro-wrinkling, leading to uneven sheet contact and disruption of sheet architecture. In contrast, neural sheets manipulated automatically retained their flat form without deformation or rupture, allowing β-III tubulin-positive neurons to remain associated with GFAP-positive astrocytes (Fig. 6b). To evaluate repeatability, 10 independent transfer trials were performed. Eight of ten neural sheets were successfully transferred and adhered to the target substrate, maintaining more than 60% of their original surface area after attachment. The distribution of surface area ratios is summarized in Supplementary Fig. S1, demonstrating consistent and gentle transfer performance.

## Discussion

This study presents an automated manipulator capable of transferring fragile cell sheets by integrating thermo-responsive materials with programmable motion control. A critical requirement for successful operation is the rapid and precise control of hydrogel shrinkage and expansion between 25 and 35 °C, synchronized with manipulator movements. This was achieved through a heat-resistor-based microheater that delivers localized heating to the microchanneled PNIPAAm hydrogel. The customized LabVIEW DAQ system enabled real-time toggling of heating states, ensuring that thermal actuation was precisely coordinated with mechanical positioning. Also, the Z-location apparatus made to decouple vertical positioning from direct motor drive reduced mechanical stress by applying only a uniform, passive load to the sheet surface. By coupling these functions, the manipulator achieved repeatable gripping and releasing cycles with minimal operator input.

The ability to automate cell sheet transfer addresses a significant challenge in tissue engineering. Manual methods, such as using tweezers or pipettes, frequently induce folding, tearing, or wrinkling of delicate sheets, thereby compromising morphology and reducing reproducibility. In contrast, our results demonstrate that the manipulator provides automated, repeatable, and gentle handling of sheets, reducing handling viability and preserving the structural integrity of transferred sheets.

Importantly, the biological results indicate that manipulator-mediated transfer not only preserves sheet architecture but also enables functional interactions in co-culture systems. In particular, endothelial cells beneath neural sheets displayed vasculature-like structures, suggesting active endothelial–neural communication. This observation implies that intact sheet transfer is essential for supporting physiologically relevant interactions, which are often disrupted by damage or deformation during manual handling. Thus, beyond technical improvements, the manipulator may facilitate more biologically representative co-culture models.

While the present design demonstrates significant improvements over conventional methods (De et al. 2007; Tadakuma et al. 2013; Leangarun et al. 2016; Osada et al. 2020; Zurina et al. 2023), it also differs from existing manual cell sheet handling approaches. Compared to these, the present platform integrates automation with mechanical compliance to minimize direct human handling. The Z-apparatus decouples vertical motion from the motor drive, providing passive compliance that accommodates variations in substrate thickness without requiring precise positional calibration. This design enables the application of a uniform, low-magnitude contact force, thereby reducing mechanical stress and preventing tearing or deformation of fragile cell sheets during manipulation. Several considerations remain for future development. The heating–cooling cycle, although rapid, may be further optimized by integrating feedback sensors for closed-loop thermal control. The passive Z-axis compliance ensures consistent loading but may limit throughput when larger or multilayer constructs are required; future designs could incorporate adjustable load calibration to expand versatility. In addition, long-term viability studies and functional assays in multilayered constructs will be necessary to evaluate the manipulator’s potential for tissue engineering and regenerative medicine applications (Lin et al. 2013; Cerqueira et al. 2013; Leangarun et al. 2016; Ryu et al. 2020; Osada et al. 2020)^,^

Overall, these results confirm that the automatic electrothermal manipulator enables automated, gentle, and reproducible handling of fragile biological samples, thereby supporting its utility for constructing layered co-cultures and potentially advancing scalable tissue engineering strategies. We also envisage that the manipulator is also capable of transferring thin polymeric substrates, such as polyimide films. These materials are widely used as platforms for biosensors and flexible biomedical devices in both in vitro and in vivo applications (Lee et al. 2016; Zhang et al. 2023; Imai et al. 2023). The gentle, reversible adhesion mechanism of the hydrogel allows precise positioning of such thin films without folding or damage, enabling their integration with living tissues or delicate culture substrates. This expands the utility of the manipulator beyond cell sheet engineering to include lamination or stacking of thin-film biosensors and biomedical devices, offering new opportunities for hybrid bioelectronic–tissue systems.

## Supplementary Information

Below is the link to the electronic supplementary material.

Supplementary Material 1 (DOCX 30.5 KB)

Supplementary Material 2 (MP4 16.4 MB)

Supplementary Material 3 (MP4 132 MB

## Data Availability

No datasets were generated or analysed during the current study.
