# Supplementary material for: Automation of electrothermal cell sheet manipulator for seamless tissue assembly and handling
Source: Biomed Microdevices. 2025 Nov 21;27(4):52. doi: 10.1007/s10544-025-00781-y (PMC12638413; doi:10.1007/s10544-025-00781-y)
Supplement: Supplementary file 1 — Supplementary Material 1 (DOCX 30.5 KB) [file 10544_2025_781_MOESM1_ESM.docx]

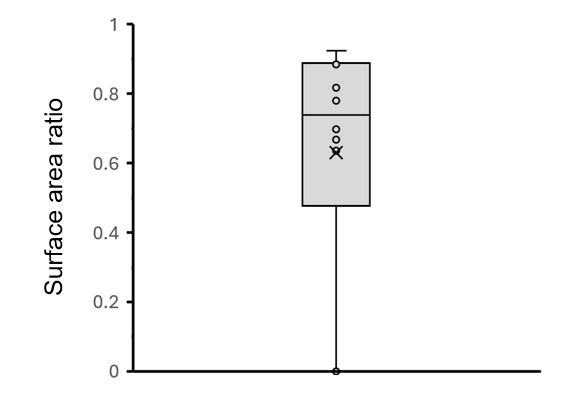


Fig. S1 Quantitative assessment of the repeatability of automated cell sheet transfer. Box plot showing the ratio of the attached surface area after transferring to the original surface area (attached/original) for ten independent trials (N = 10). The median, interquartile range, and individual data points are displayed, demonstrating consistent and reproducible transfer performance.
